# Editorial: Leptin in Physiology and Disease

**DOI:** 10.3389/fphys.2022.950686

**Published:** 2022-07-08

**Authors:** Deanne H. Hryciw, Harbindar Jeet Singh

**Affiliations:** ^1^ School of Environment and Science, Griffith University, Nathan, QLD, Australia; ^2^ Centre for Planetary Health and Food Security, Griffith University, Nathan, QLD, Australia; ^3^ Institute for Health and Sport, Victoria University, Melbourne, VIC, Australia; ^4^ Department of Physiology, School of Medical Sciences, Universiti Teknologi MARA, Selangor, Malaysia

**Keywords:** leptin, pathophysiology, organs and organ systems, disease, health

Since the discovery of leptin, a product of the *ob* gene, in 1994, there has been a large amount of research identifying the role of this hormone in the maintenance of normal physiology and in disease states. Specifically, the adipokine leptin plays a significant role in the maintenance of homeostasis through modulating cell signalling pathways involved in energy balance and food intake. More recent research has investigated the role of leptin in pathways not linked to satiety, with researchers demonstrating that leptin contributes to other physiological processes including inflammation, respiratory function, bone metabolism, and development. Furthermore, this research has been extended to demonstrate leptin’s mechanistic links to pathologies associated with diseases, particularly those related to obesity.

This Research Topic aims to explore the diverse roles that leptin and its signalling pathways play in a number of biological systems, as well as disease states. The Research Topic contains 2 review articles, 1 original manuscript, and 1 hypothesis and theory manuscript. Collectively, they demonstrate the varied roles leptin plays in maintaining homeostasis.

SARS-CoV 2 coronavirus disease 2019 (COVID-19) has made a significant impact worldwide, with pathologies associated with COVID-19 infection affecting a number of key organs. The review by Bruno et al. discusses the role of leptin as an immune regulator, and its potential links to pathologies associated with COVID-19 infection. Obesity, which leads to an increase in circulating concentrations of leptin, is a proinflammatory state, with obese individuals having an increased risk of pulmonary infections. Bruno et al. describes the important role of the microbiota in modulating lung function via the “gut-lung axis” in addition to impaired immunity. Bruno et al. propose that leptin and microbiota control the gut-lung axis, which may contribute to the pathophysiological changes in the lung associated with COVID-19 infection. Independent of the direct action between COVID-19 and angiotensin-converting enzyme 2 and transmembrane serine protease 2, compromised immune function due to dysfunctional leptin signalling may exacerbate the pathological changes associated with COVID-19.

The review by LeDuc et al. discusses the prenatal and perinatal roles of leptin in the development of the nervous system, specifically the neuronal pathways that program adiposity in later life. During development there is a leptin peak, which is critical for the completion of organogenesis. LeDuc et al. highlighted the role of the nutritional environment in controlling leptin signaling as it relates to neurodevelopment. These authors suggest that modulation of leptin signalling in the intrauterine environment may be a potential therapeutic in reducing the risk of developing obesity in adulthood.

The diversity of leptin’s role in normal physiology is extended to bone dynamics and remodelling. The third review of this Research Topic explores the hypothesis that leptin controls bone dynamics via the vesicle trafficking protein endospanin. Londraville et al. concede that the previous studies with contradictory results linking leptin and bone dynamics observed in rodent models are likely due to the employment of models which rely on the removal of leptin signalling. Londraville et al. promote the use of zebrafish in exploring the role of leptin signalling in bone dynamics, and in particular in understanding the role of leptin resistance in obesity associated bone remodelling.

The final manuscript of this Research Topic is an original research manuscript by Ciriello et al., investigating the underlying mechanism that links obstructive sleep apnoea and obesity. Ciriello et al. exposed Sprague-Dawley rats to either chronic intermittent hypoxia (CIH), or normoxic control conditions. CIH is an established model for chronic sleep apnoea. In these animals, CIH rats were found to have reduced locomotor activity and food conversion efficiency. Additionally, the CIH rats had increased food and water intake and a higher body weight compared to normoxic controls. Basal plasma concentrations of leptin were significantly elevated in CIH rats even before an evident increase in body weight, and acute exogenous leptin injection failed to control satiety in CIH rats, which was observed in normoxic controls. Furthermore, leptin receptor expression was reduced in the hypothalamic arcuate nucleus, as was the expression of pro-opiomelanocortin protein expression in CIH rodents. This rodent model suggests that there may be a mechanistic link between sleep apnoea and obesity via altered perturbations in leptin resistance promoting an increase in body weight. Their findings add another aspect to the widely held view of obesity leading to sleep apnoea.

